# Interim Estimates of 2017–18 Seasonal Influenza Vaccine Effectiveness — United States, February 2018

**DOI:** 10.15585/mmwr.mm6706a2

**Published:** 2018-02-16

**Authors:** Brendan Flannery, Jessie R. Chung, Edward A. Belongia, Huong Q. McLean, Manjusha Gaglani, Kempapura Murthy, Richard K. Zimmerman, Mary Patricia Nowalk, Michael L. Jackson, Lisa A. Jackson, Arnold S. Monto, Emily T. Martin, Angie Foust, Wendy Sessions, LaShondra Berman, John R. Barnes, Sarah Spencer, Alicia M. Fry

**Affiliations:** ^1^Influenza Division, National Center for Immunization and Respiratory Diseases, CDC; ^2^Marshfield Clinic Research Institute, Marshfield, Wisconsin; ^3^Baylor Scott & White Health, Texas A&M University Health Science Center College of Medicine, Temple, Texas; ^4^University of Pittsburgh Schools of the Health Sciences and University of Pittsburgh Medical Center, Pittsburgh, Pennsylvania; ^5^Kaiser Permanente Washington Health Research Institute, Seattle, Washington; ^6^University of Michigan, Ann Arbor, Michigan.

In the United States, annual vaccination against seasonal influenza is recommended for all persons aged ≥6 months (*1*). During each influenza season since 2004–05, CDC has estimated the effectiveness of seasonal influenza vaccine to prevent laboratory-confirmed influenza associated with medically attended acute respiratory illness (ARI). This report uses data from 4,562 children and adults enrolled in the U.S. Influenza Vaccine Effectiveness Network (U.S. Flu VE Network) during November 2, 2017–February 3, 2018. During this period, overall adjusted vaccine effectiveness (VE) against influenza A and influenza B virus infection associated with medically attended ARI was 36% (95% confidence interval [CI] = 27%–44%). Most (69%) influenza infections were caused by A(H3N2) viruses. VE was estimated to be 25% (CI = 13% to 36%) against illness caused by influenza A(H3N2) virus, 67% (CI = 54%–76%) against A(H1N1)pdm09 viruses, and 42% (CI = 25%–56%) against influenza B viruses. These early VE estimates underscore the need for ongoing influenza prevention and treatment measures. CDC continues to recommend influenza vaccination because the vaccine can still prevent some infections with currently circulating influenza viruses, which are expected to continue circulating for several weeks. Even with current vaccine effectiveness estimates, vaccination will still prevent influenza illness, including thousands of hospitalizations and deaths. Persons aged ≥6 months who have not yet been vaccinated this season should be vaccinated.

Methods used by the U.S. Flu VE Network have been published previously (*2*). At five study sites,* patients aged ≥6 months seeking outpatient medical care for an ARI with cough within 7 days of illness onset were enrolled. Study enrollment began after local surveillance identified increasing weekly influenza activity or one or more laboratory-confirmed cases of influenza per week for 2 consecutive weeks. Patients were eligible for enrollment if they 1) were aged ≥6 months on September 1, 2017, and thus were eligible for vaccination; 2) reported an ARI with cough with onset ≤7 days earlier; and 3) had not been treated with influenza antiviral medication (e.g., oseltamivir) during this illness. After obtaining informed consent from patients or from parents or guardians for their children, participants or their proxies were interviewed to collect demographic data, information on general and current health status and symptoms, and 2017–18 influenza vaccination status. Nasal and oropharyngeal swabs (or nasal swabs alone for children aged <2 years) were collected to obtain respiratory specimens; nasal and oropharyngeal swabs were placed together in a single cryovial with viral transport medium. Specimens were tested at U.S. Flu VE Network laboratories using CDC’s real-time reverse transcription polymerase–chain reaction (rRT-PCR) protocol for detection and identification of influenza viruses. Participants (including children aged <9 years, who require 2 vaccine doses during their first vaccination season) were considered vaccinated if they received ≥1 dose of any seasonal influenza vaccine ≥14 days before illness onset, according to medical records and registries (at the Wisconsin site); medical records and self-report (at the Washington site); or self-report only (at the Michigan, Pennsylvania, and Texas sites). VE against all influenza virus types combined and against viruses by type/subtype was estimated as 100% x (1 - odds ratio).^†^ Estimates were adjusted for study site, age group, sex, race/ethnicity, self-rated general health, number of days from illness onset to enrollment, and week of illness (3-week intervals) using logistic regression. Interim VE estimates for the 2017–18 season were based on patients enrolled through February 3, 2018.

Among the 4,562 children and adults with ARI enrolled at the five study sites from November 2, 2017, through February 3, 2018, a total of 1,712 (38%) tested positive for influenza virus by rRT-PCR, including 1,392 (81%) influenza A viruses and 323 (19%) influenza B viruses (Table 1). Among 1,340 subtyped influenza A viruses, 1,143 (85%) were A(H3N2) viruses and 208 (16%) were A(H1N1)pdm09 viruses. Most (98%) influenza B viruses belonged to the B/Yamagata lineage. The proportion of patients with influenza differed by study site, sex, age group, race/ethnicity, self-rated health status, and interval from illness onset to enrollment (Table 1). The percentage of patients who were vaccinated ranged from 45% to 59% among study sites and differed by sex, age group, race/ethnicity, and self-rated health status.

**TABLE 1:** Selected characteristics for 4,562 enrolled outpatients with medically attended acute respiratory illness and cough, by influenza test result status and seasonal influenza vaccination status — U.S. Influenza Vaccine Effectiveness Network, United States, November 2, 2017–February 3, 2018

Characteristic	Test result status		p-value^†^	Vaccination status*		p-value^†^
	Influenza-positive	Influenza-negative		Vaccinated		
	No. (%)	No. (%)		No. enrolled	No. (%) vaccinated	
**Overall**	**1,712 (38)**	**2,850 (62)**	**—**	**4,562**	**2,259 (50)**	**—**
**Study site**						
Michigan	264 (35)	491 (65)	<0.001	755	422 (56)	<0.001
Pennsylvania	330 (41)	480 (59)		810	376 (46)	
Texas	572 (42)	806 (58)		1,378	614 (45)	
Washington	195 (27)	518 (73)		713	420 (59)	
Wisconsin	351 (39)	555 (61)		906	427 (47)	
**Sex**						
Male	735 (39)	1,133 (61)	0.03	1,868	865 (46)	<0.001
Female	977 (36)	1,717 (64)		2,694	1,394 (52)	
**Age group (yrs)**						
6 mos–8	359 (33)	739 (67)	<0.001	1,098	535 (49)	<0.001
9–17	288 (49)	300 (51)		588	204 (35)	
18–49	561 (36)	989 (64)		1,550	642 (41)	
50–64	288 (39)	454 (61)		742	436 (59)	
≥65	216 (37)	368 (63)		584	442 (76)	
**Race/Ethnicity** ^§^						
White	1,169 (37)	2,020 (63)	0.004	3,189	1,659 (52)	<0.001
Black	161 (43)	218 (58)		379	150 (40)	
Other race	144 (33)	287 (67)		431	217 (50)	
Hispanic	231 (42)	317 (58)		548	225 (41)	
**Self-rated health status**						
Fair or poor	75 (31)	168 (69)	<0.001	243	135 (56)	<0.001
Good	377 (35)	695 (65)		1,072	559 (52)	
Very good	618 (36)	1,087 (64)		1,705	875 (51)	
Excellent	639 (42)	898 (58)		1,537	687 (45)	
**Illness onset to enrollment (days)**						
<3	856 (48)	940 (52)	<0.001	1,796	866 (48)	0.23
3–4	589 (35)	1,082 (65)		1,671	829 (50)	
5–7	267 (24)	828 (76)		1,095	564 (52)	
**Influenza test result** ^¶^						
Negative	—	2,850	—	2,850	1,518 (53)	—
Influenza B positive	323	—	—	323	132 (41)	—
B/Yamagata	260	—	—	260	112 (43)	—
B/Victoria	5	—	—	5	2 (40)	—
B lineage pending	58	—	—	58	18 (31)	—
Influenza A positive	1,392	—	—	1,392	610 (44)	—
A(H1N1)pdm09	208	—	—	208	60 (29)	—
A(H3N2)	1,143	—	—	1,143	530 (46)	—
A subtype pending	52	—	—	52	23 (44)	—

* Defined as having received ≥1 dose of influenza vaccine ≥14 days before illness onset. A total of 102 participants who received the vaccine ≤13 days before illness onset were excluded from the study sample.

^†^ The chi-square statistic was used to assess differences between the numbers of persons with influenza-negative and influenza-positive test results, in the distribution of enrolled patient and illness characteristics, and in differences between groups in the percentage vaccinated.

^§^ Enrollees were categorized into one of four mutually exclusive racial/ethnic populations: white, black, other race, and Hispanic. Persons identifying as Hispanic might have been of any race. Persons identifying as white, black, or other race were non-Hispanic. Race/ethnicity data were missing for 15 enrollees.

^¶^ Fourteen patients had coinfection with influenza A and influenza B, making the sum 1,726, or 14 greater than the total number of influenza-positive patients.

Among ARI patient participants, 43% of those with influenza had received the 2017–18 seasonal influenza vaccine, compared with 53% of influenza-negative participants (Table 2). After adjusting for study site, age group, sex, race/ethnicity, self-rated general health, number of days from illness onset to enrollment, and week of illness onset (3-week intervals), VE against medically attended ARI caused by all influenza virus types combined was 36% (CI = 27%–44%). VE for all ages was 25% (CI = 13% to 36%) against medically attended ARI caused by A(H3N2) virus infection, 67% (CI = 54%–76%) against influenza A(H1N1)pdm09 virus infection, and 42% (CI = 25%–56%) against influenza B virus infection. VE point estimates against medically attended influenza for all virus types varied by age group; statistically significant protection against medically attended influenza was found among children aged 6 months through 8 years (VE = 59%; CI = 44%–69%) and adults aged 18–49 years (VE = 33%; CI = 16%–47%), whereas no statistically significant protection was observed in other age groups.

**TABLE 2:** Number and percentage receiving 2017–18 seasonal influenza vaccine among 4,562 enrolled outpatients with medically attended acute respiratory illness and cough, by influenza test result status, age group, and vaccine effectiveness against all influenza A and B and against virus types A(H3N2), A(H1N1)pdm09 and B — U.S. Influenza Vaccine Effectiveness Network, United States, November 2, 2017–February 3, 2018

Influenza type/Age group	Test result status				Vaccine effectiveness*	
	Influenza-positive		Influenza-negative		Unadjusted	Adjusted
	Total	No. (%) vaccinated	Total	No. (%) vaccinated	% (95% CI)	% (95% CI)
**Influenza A and B**						
**Overall**	**1,712**	**741 (43)**	**2,850**	**1,518 (53)**	**33 (24 to 41)**	**36 (27 to 44)^†^**
**Age group (yrs)**						
6 mos–8	359	127 (35)	739	408 (55)	56 (42 to 66)	59 (44 to 69)^†^
9–17	288	100 (35)	300	104 (35)	0 (-41 to 29)	5 (-38 to 34)
18–49	561	198 (35)	989	444 (45)	33 (17 to 46)	33 (16 to 47)^†^
50–64	288	159 (55)	454	277 (61)	21 (-6 to 42)	17 (-15 to 40)
≥65	216	157 (73)	368	285 (78)	23 (-14 to 47)	18 (-25 to 47)
**Influenza A(H3N2)**						
**Overall**	**1,143**	**530 (46)**	**2,850**	**1,518 (53)**	**24 (13 to 34)**	**25 (13 to 36)^†^**
**Age group (yrs)**						
6 mos–8	200	79 (40)	739	408 (55)	47 (27 to 61)	51 (29 to 66)^†^
9–17	203	75 (37)	300	104 (35)	-10 (-60 to 24)	-8 (-62 to 29)
18–49	395	155 (39)	989	444 (45)	21 (-1 to 37)	20 (-4 to 38)
50–64	198	115 (58)	454	277 (61)	11 (-24 to 37)	12 (-26 to 39)
≥65	147	106 (72)	368	285 (78)	25 (-16 to 51)	17 (-35 to 49)
**Influenza A(H1N1)pdm09**						
**Overall**	**208**	**60 (29)**	**2,850**	**1,518 (53)**	**64 (52 to 74)**	**67 (54 to 76)^†^**
**Age group (yrs)**						
<18	105	22 (21)	1,039	512 (49)	73 (56 to 83)	78 (63 to 87)^†^
18–64	84	26 (31)	1,443	721 (50)	55 (28 to 72)	51 (20 to 70)^†^
≥65	19	12 (63)	368	285 (78)	50 (-31 to 81)	34 (-96 to 78)
**Influenza B**						
**Overall**	**323**	**132 (41)**	**2,850**	**1,518 (53)**	**39 (23 to 52)**	**42 (25 to 56)^†^**
**Age group (yrs)**						
<18	127	46 (36)	1,039	512 (49)	42 (14 to 60)	36 (1 to 58)^†^
18–64	151	53 (35)	1,443	721 (50)	46 (23 to 62)	50 (28 to 66)^†^
≥65	45	33 (73)	368	285 (78)	20 (-62 to 60)	25 (-62 to 66)

**Abbreviation:** CI = confidence interval.

* Vaccine effectiveness was estimated as 100% x (1 - odds ratio [ratio of odds of being vaccinated among outpatients with influenza-positive test results to the odds of being vaccinated among outpatients with influenza-negative test results]); odds ratios were estimated using logistic regression.

^†^ Statistically significant at the p<0.05 level.

As of February 3, 2018, a total of 257 influenza A(H3N2) viruses from U.S. Flu VE Network participants had been characterized by CDC; 240 (93%) belonged to either genetic group 3C.2a (226 viruses) or the related subgroup 3C.2a1 (14), whereas 17 (7%) belonged to group 3C.3a. Genetic group 3C.2a includes the A/Hong Kong/4801/2014 reference virus representing the A(H3N2) component of the 2017–18 Northern Hemisphere influenza vaccines (*3*).

## Discussion

Early and widespread influenza activity during the 2017–18 influenza season provided the opportunity to estimate interim VE against several circulating influenza viruses, including the predominant A(H3N2) virus. These interim estimates reflect ongoing challenges with the A(H3N2) vaccine component since the 2011–12 season. The interim estimate of 25% VE against A(H3N2) viruses this season indicates that vaccination provided some protection, in contrast to recently reported, nonsignificant interim estimates of 17% from Canada and 10% from Australia (*4*,*5*) and is similar to final (32%) VE estimates in the United States against A(H3N2) viruses during 2016–17^§^ (*6*). However, among children aged 6 months through 8 years, the interim estimates against any influenza and A(H3N2) virus infection were higher; the risk for A(H3N2) associated medically-attended influenza illness was reduced by more than half (59%) among vaccinated children. Also, with interim VE estimates of 67% and 42% against influenza A(H1N1)pdm09 and B viruses, respectively, vaccination provided substantial protection against circulating A(H1N1)pdm09 viruses, as well as moderate protection against influenza B viruses predominantly belonging to the B/Yamagata lineage, the second influenza type B component included in quadrivalent vaccines. CDC continues to recommend influenza vaccination while influenza viruses are circulating in the community; several more weeks of influenza activity are likely. Influenza vaccination has prevented thousands of hospitalizations during previous seasons when influenza A(H3N2) viruses were predominant, including during the 2014–15 season when interim VE estimates were similar to those reported here. Appropriate use of influenza antiviral medications for treatment of severely ill persons or persons at high risk for complications from influenza who develop influenza symptoms is important, especially among older adults, who currently have the highest hospitalization rates (*3*).

VE estimates against A(H3N2) viruses have been lower than estimates against A(H1N1)pdm09 and B viruses for several years (*7*). Although there is no definitive evidence for antigenic drift of viruses circulating this season compared with cell culture–propagated reference viruses representing the A(H3N2) vaccine component (*3*), challenges with antigenic characterization of recent A(H3N2) viruses, many of which could not be characterized using traditional hemagglutination inhibition assays, have required the use of additional virus neutralization assays to assess antigenic characteristics. Multiple factors might be contributing to the reported VE against A(H3N2) viruses this season. Immune responses to vaccination differ by age and previous infection or vaccination history and can affect vaccine protection; higher VE against A(H3N2) viruses among young children suggests that vaccination might provide better protection against circulating A(H3N2) viruses to this age group. Also, genetic changes in the vaccine virus hemagglutinin protein that arise during passage in eggs might result in a vaccine immune response that is less effective against circulating viruses (*8*,*9*). Human serologic data indicate decreased inhibition of circulating cell culture–propagated A(H3N2) viruses compared with egg-propagated viruses among persons vaccinated with egg-based vaccines.^¶^ Additional studies are needed to assess whether VE against circulating A(H3N2) viruses varies by vaccine type, including comparisons between egg-based and non–egg-based vaccines. CDC will continue to monitor VE through the remainder of the season and is investigating these factors. In addition, many efforts are under way to improve selection and development of candidate vaccine viruses that are optimal for vaccine production and provide protection against a majority of circulating viruses.

These interim VE estimates underscore the need for influenza antiviral treatment for any patient with suspected or confirmed influenza who is hospitalized, has severe or progressive illness, or is at high risk for complications from influenza, regardless of vaccination status or results of rapid, point-of-care influenza diagnostic tests.** CDC recommends antiviral medications as an adjunct to vaccination, and their potential public health benefit is increased in the context of low VE. A CDC health update issued December 27, 2017, regarding treatment with antiviral medications is available at https://emergency.cdc.gov/han/han00409.asp. Clinicians should be aware that influenza activity is widespread, and influenza should be considered as a possible diagnosis in all patients with acute respiratory illness.

The findings in this report are subject to at least four limitations. First, vaccination status included self-report at four of five sites. End-of-season VE estimates based on updated documentation of vaccination status might differ from interim estimates. Second, information from medical records and immunization registries is needed to evaluate VE by vaccine type and for fully vaccinated versus partially vaccinated children, as well as to evaluate the effects of previous season vaccination and timing of vaccination; end-of-season analysis of VE by vaccine type and effects of partial or previous season vaccination is planned. Third, an observational study design has greater potential for confounding and bias relative to randomized clinical trials. However, the test-negative design is widely used in VE studies and has been used by the U.S. Flu VE Network to estimate VE for previous influenza seasons. Finally, small sample sizes in some age groups resulted in wide confidence intervals, and end-of-season VE estimates could change as additional patient data become available or if there is a change in circulating viruses late in the season. It is also important to note that the VE estimates in this report are limited to the prevention of outpatient medical visits rather than more severe illness outcomes, such as hospitalization or death; data from studies measuring VE against more severe outcomes will be available at a later date.

Annual monitoring of VE supports ongoing efforts to improve influenza vaccines. Although more effective vaccines are needed, vaccination prevents a substantial burden of influenza-related illness annually. During the 2014–15 season, when VE against medically attended illness caused by any influenza virus was less than 20%, vaccination was estimated to prevent 11,000–144,000 influenza-associated hospitalizations and 300–4,000 influenza-associated deaths (https://www.cdc.gov/flu/about/disease/2014-15.htm). Small increases in VE can substantially affect the number of hospitalizations prevented during a severe season (*10*). Although interim estimates suggest that vaccination has prevented some influenza-related illness this season, influenza vaccines with improved effectiveness are needed to substantially reduce the incidence of disease.

**Summary**

**What is already known about this topic?**

Effectiveness of seasonal influenza vaccine can vary by season and has generally been higher against influenza A(H1N1)pdm09 and B viruses than against A(H3N2) viruses.

**What is added by this report?**

So far this season, influenza A(H3N2) viruses have predominated, but other influenza viruses are also circulating. Based on data from 4,562 children and adults with acute respiratory illness enrolled during November 2, 2017–February 3, 2018, at five study sites with outpatient medical facilities in the United States, the overall estimated effectiveness of the 2017–18 seasonal influenza vaccine for preventing medically attended, laboratory-confirmed influenza virus infection was 36%.

**What are the implications for public health practice?**

CDC continues to monitor influenza vaccine effectiveness. Influenza vaccination is still recommended; vaccination reduces the risk for influenza illnesses and serious complications. Treatment with influenza antiviral medications, where appropriate, is especially important this season.

## Footnotes

* The U.S. Flu VE Network sites and the dates enrollment began are as follows: Kaiser Permanente Washington (Seattle, Washington) (November 27, 2017); Marshfield Clinic Research Institute (Marshfield, Wisconsin) (December 26, 2017); University of Michigan School of Public Health (the School of Public Health partnered with the University of Michigan Health System, Ann Arbor, and the Henry Ford Health System, Detroit, Michigan) (December 4, 2017); University of Pittsburgh Schools of the Health Sciences (the Schools of the Health Sciences partnered with the University of Pittsburgh Medical Center, Pittsburgh, Pennsylvania) (November 29, 2017); and Baylor Scott & White Health, Texas A&M University Health Science Center College of Medicine (Temple, Texas) (November 2, 2017).

† 100% x (1 - odds ratio [ratio of odds of being vaccinated among outpatients with influenza-positive test results to the odds of being vaccinated among outpatients with influenza-negative test results]).

§ https://www.cdc.gov/vaccines/acip/meetings/downloads/slides-2017-06/flu-03-ferdinands.pdf.

¶ http://apps.who.int/iris/bitstream/10665/259275/1/WER9242.pdf?ua=1.

** A complete summary of guidance for antiviral use is available at https://www.cdc.gov/flu/professionals/antivirals/summary-clinicians.htm. Groups at high risk for influenza complications include the following: children aged <2 years; adults aged ≥65 years; persons with chronic pulmonary conditions (including asthma); persons with cardiovascular disease (except hypertension alone); persons with renal, hepatic, or hematologic (including sickle cell) disease; persons with metabolic disorders (including diabetes mellitus); persons with neurologic and neurodevelopmental conditions (including disorders of the brain, spinal cord, peripheral nerves and muscles, such as cerebral palsy, epilepsy [seizure disorders], stroke, intellectual disability [mental retardation], moderate to severe developmental delay, muscular dystrophy, or spinal cord injury); persons with immunosuppression, including that caused by medications or by human immunodeficiency virus infection; women who are pregnant or ≤2 weeks postpartum; persons aged <19 years who are receiving long-term aspirin therapy; American Indian/Alaska Natives; persons with morbid obesity (i.e., body-mass index ≥40); and residents of nursing homes and other chronic-care facilities.
